# Evaluation of yeast-based additives on rumen fermentation in high- and low-concentrate diets using a dual-flow continuous culture system

**DOI:** 10.1093/tas/txae169

**Published:** 2024-12-07

**Authors:** Amanda Regina Cagliari, Elaine Magnani, Kalista Eloisa Loregian, Fernanda Rigon, Ana Claudia Casagrande, Bruna Roberta Amancio, Charles Marcon Giacomelli, Juliana Bueno da Silva, Veronica Lisboa Santos, Marcos Inacio Marcondes, Renata Helena Branco, Pedro Del Bianco Benedeti, Eduardo Marostegan de Paula

**Affiliations:** Department of Animal Science, Universidade do Estado de Santa Catarina, Chapecó, Brazil; Institute of Animal Science, Beef Cattle Research Center, Sertãozinho, Brazil; Institute of Animal Science, Beef Cattle Research Center, Sertãozinho, Brazil; Department of Animal Science, Universidade Estadual Paulista Julio de Mesquita Filho, Jaboticabal, Brazil; Department of Animal Science, Universidade Estadual Paulista Julio de Mesquita Filho, Jaboticabal, Brazil; Department of Animal Science, Universidade do Estado de Santa Catarina, Chapecó, Brazil; Institute of Animal Science, Beef Cattle Research Center, Sertãozinho, Brazil; Department of Animal Science, Universidade do Estado de Santa Catarina, Chapecó, Brazil; Yessinergy do Brasil Agroindustrial LTDA, Campinas, Brazil; Yessinergy do Brasil Agroindustrial LTDA, Campinas, Brazil; Department of Animal Sciences, Washington State University, Pullman, WA 99163, USA; William H. Miner Agricultural Research Institute, Chazy, NY, USA; Institute of Animal Science, Beef Cattle Research Center, Sertãozinho, Brazil; Department of Animal Science, Universidade do Estado de Santa Catarina, Chapecó, Brazil; Institute of Animal Science, Beef Cattle Research Center, Sertãozinho, Brazil

**Keywords:** Feed additives, monensin, prebiotics, ruminant nutrition

## Abstract

The objective of this study was to evaluate the efficacy of using 3 yeast-based additives as an alternative to sodium monensin on rumen fermentation parameters using a dual-flow continuous fermentation system. Ten fermenters (1,223 ± 21 mL) were used in 2 simultaneous 5 × 5 Latin squares arrangement with 3 periods of 10 d each, with 7 d for diet adaptation and 3 d for sample collections. Each Latin square assigning either a low or high level of concentrate to beef cattle diets, with 5 specified treatments: Control: no additives; Blend 1: yeast culture (*Saccharomyces cerevisiae*), beta-glucans, fructooligosaccharides, galactooligosaccharides, and mannanoligosaccharides [1,600 mg/kg dry matter (DM)]; Blend 2: Beta-glucan and mannanoligosaccharide fractions from *S. cerevisiae* (1,600 mg/kg DM); Yeast Cells: hydrolyzed, inactivated, and spray-dried yeast cells (*S. cerevisiae*; 2,133 mg/kg DM); monensin (25 mg/kg DM). On days 8, 9, and 10, samples of 500 mL of solid and liquid digesta effluent were mixed, homogenized, and stored at −20 °C. Subsamples of 10 mL were collected for later determination of ammonia nitrogen (NH_3_–N) and volatile fatty acids (VFA). Diets with high-concentrate showed higher organic matter (OM) digestibility but lower crude protein and neutral detergent fiber (NDF) digestibilities (*P* < 0.01). There were no feed additive effects for DM, OM, and NDF digestibilities (*P* > 0.05). Total VFA concentration and butyrate concentration were higher for the high-concentrate diet (*P* < 0.01). Conversely, pH and concentrations of acetate and iso-butyrate were higher for the low-concentrate diet (*P* < 0.01). Treatments with Blend 1, Blend 2, and Yeast Cells had higher VFA concentrations compared to the control (*P* = 0.04). Blend 1 treatment exhibited higher propionate concentration in fermenters fed with a high-concentrate diet (*P* < 0.01). In the high-concentrate diet, Blend 1 had a lower acetate: propionate ratio compared to Control, Yeast Cells, and Blend 2 treatments (*P* < 0.01). The high-concentrate diet showed higher means for all other parameters: Microbial efficiency, N efficiency, N flow, and Bacterial N flow (*P* < 0.01). Treatments with Blend 2 and Control showed higher rumen undegradable protein N flow compared to Yeast Cells and Blend 1 treatments (*P* < 0.01). Our findings imply that yeast-based additives might be used as alternatives to monensin, improving ruminal fermentation and promoting enhanced sustainability in livestock.

## Teaser

Yeast-based additives show promise as alternatives to sodium monensin in beef cattle diets, improving rumen fermentation and supporting more sustainable livestock production.

## Introduction

The use of rumen fermentation-modulating additives aims to enhance the symbiosis between ruminal microorganisms and their host, improving the utilization of nutrients from provided feed and consequently enhancing animal performance (Ban and Guan, 2021). Among various substances, monensin has been the most studied and utilized additive to increase feed efficiency in cattle (Baggio et al., 2023). However, this ionophore has the potential to leave residues in animal-derived products and may induce resistance in certain bacterial strains (Alexopoulos et al., 2004). Therefore, there is a need to find alternative additives with similar ruminal fermentation-modulating potential that do not induce bacterial resistance. This is crucial because the mechanism by which ionophores act on ruminal bacteria is linked to resistance factors present in the cell wall. This structure is responsible for maintaining the chemical balance between the intracellular and extracellular environments (Ballou et al., 2019).

The use of yeast-based prebiotics aims to create a stable environment for the development of beneficial bacteria in the rumen (Chaucheyras-Durand et al., 2012). Moreover, these natural additives have the potential to enhance rumen fermentation and increase feed degradation, resulting in better nutrient utilization by microorganisms and improved animal performance, without risks to human health (Jia et al., 2018). Yeast-based prebiotics do not act like antibiotics; instead, they provide favorable conditions for the growth of specific bacterial groups (Mcginn et al., 2004). The mechanism of action of these prebiotics involves removing oxygen (O_2_) from the environment, thus promoting the growth of anaerobic bacteria (Chaucheyras-Durand et al., 2012). Another characteristic of these additives is their cell wall composition, which consists of polysaccharides capable of interacting with bacteria, binding to them, and consequently preventing harmful microorganisms from attaching to the gastrointestinal tract of animals (Kogan and Kocher, 2007; Gaggia et al., 2010).

Beta-glucans, galactooligosaccharides (GOS), and fructooligosaccharides (FOS) are natural bioactive compounds with antibacterial and metabolic regulatory effects that could enhance ruminal fermentation modulation (Kanakupt et al., 2011; Krüger and van der Werf, 2018; Li et al., 2019; Smiricky-Tjardes et al., 2003). These molecules have demonstrated the ability to modulate volatile fatty acid (VFA) production and profile, as well as improve fiber digestibility (Grove et al., 2006; Kanakupt et al., 2011; Smiricky-Tjardes et al., 2003). However, further research is required to evaluate these compounds, both with and without yeast cultures, in comparison to monensin, to determine the optimal dosage for using these yeast-based additives in ruminant feeding. In this regard, a study conducted by Cagliari et al. (2023), which used an in vitro gas production system, demonstrated that yeast-based additives have the potential to replace monensin as a rumen fermentation modulator. In this study, multiple levels of 3 prebiotics were tested, allowing identification of the most effective levels for each additive and establishing a baseline for subsequent prebiotic evaluations. Furthermore, the findings of this study indicate that some yeast-based additives influenced propionate concentrations, while others influenced acetate concentrations. Thus, we hypothesized that their effects could differ in high- and low-concentrate diets. The influence of concentrate inclusion on ruminal fermentation dynamics, metabolic byproduct composition, and fermentation patterns varies significantly based on the levels of concentrate in the diet (Huntington et al., 2006; Lechartier and Peyraud, 2011). Therefore, the next step would involve assessing these additives in more sophisticated rumen fermentation simulation systems and studying their impacts on diets with varying concentrate levels.

The aim of this study was to assess the effectiveness of 3 yeast-based additives as substitutes for sodium monensin in influencing rumen fermentation parameters of diets with high and low levels of concentrate, using a dual-flow continuous fermentation system. Our hypothesis posited that incorporating yeast-based additives could serve as a viable replacement for sodium monensin in beef cattle diets across different concentrate levels. We anticipated that this substitution would result in similar nutrient digestibility, VFA, ammonia nitrogen (NH_3_–N), pH, and nitrogen metabolism.

## Materials and Methods

### Previous Study

A previous study was conducted to assess the effectiveness of using 3 yeast-based additives as alternatives to sodium monensin on ruminal fermentation parameters in a gas production system (Cagliari et al., 2023). The additives used were: Blend 1, a prebiotic composed of *Saccharomyces cerevisiae* yeast culture, beta-glucans, fructooligosaccharides, galactooligosaccharides, and mannanoligosaccharides (Golf; Yessinergy LTDA, Campinas, SP, Brazil); Blend 2, a prebiotic mixture composed of beta-glucan and mannanoligosaccharide fractions from *S. cerevisiae* (GlucanMos; Yessinergy LTDA); and Yeast Cells, consisting of hydrolyzed, inactivated, and spray-dried yeast cells (*S. cerevisiae;* BioHydro; Yessinergy LTDA). The study evaluated 5 levels of inclusion for each additive [0, 533, 1,067, 1,600, and 2,133 mg/kg of dry matter (DM)]. Subsequently, the dosages that showed promising results were selected for evaluation in the present study.

### Experimental Design

The experimental design consisted of 2 simultaneous 5 × 5 Latin squares. Each Latin square design involved assigning either a low or high level of concentrate to the beef cattle diets (Table 1). Within each Latin square, the 5 treatments used were Control: Diet without additives; Blend 1 (1,600 mg/kg DM); Blend 2 (1,600 mg/kg DM); Yeast Cells: (2,133 mg/kg DM); Monensin: Rumensin (25 mg/kg DM). To evaluate these treatments, 10 fermenter jars were used in a dual-flow continuous fermentation system (ENG-RM-1 model; Engco LTDA, Piracicaba, SP), with an average individual volume of 1,297.13 mL (±32.82 mL), adapted from the model proposed by (Hoover et al., 1976). The experimental periods lasted for 10 d, with 7 d for adaptation and 3 d for sampling.

**Table 1.** Ingredients and chemical composition of experimental diets

Item	Concentrate level	
	Low	High
Ingredient, g/kg		
Tifton 85 hay	800	200
Dry ground corn	80.0	560
Soybean meal	70.0	105
Citrus pulp	25.0	110
Mineral mixture^†^	25.0	25.0
Composition, g/kg DM		
Dry matter, g/kg	868	894
Organic matter	801	832
Crude protein	126	134
Ether extract	19.0	30.7
Neutral detergent fiber	624	282

^†^Guaranteed Levels per kilogram: Calcium (min/max): 160.00/170.00 g, Phosphorus (min): 80.00 g, Magnesium (min): 8.00 g, Sulfur (min): 22.00 g, Sodium (min): 120.00 g, Selenium (min): 18.00 g, Copper (min): 1,000.00 mg, Zinc (min): 3,600.00 mg, Manganese (min): 700.00 mg, Iodine (min): 80.00 mg, Cobalt (min): 80.00 mg.

### Experimental Procedures

The fermenters were inoculated with rumen fluid collected from 6 cannulated Nellore steers (average weight of 710 ± 20 kg) comprising 3 from a high-concentrate group and 3 from a low-concentrate group. Prior to collection, the animals were fed diets matching their respective concentrate level for a 14-d period. The high-concentrate diet consisted of 60% corn silage and 40% concentrated feed (including ground corn, soybean meal, and mineral mix) as the basal diet, while the low-concentrate diet primarily consisted of Brachiaria pasture supplemented with mineral salt.

Rumen fluid collection took place approximately 2 h before the animals’ daily feeding, following the recommendations of Yáñez-Ruiz et al. (2016). For each incubation, the collected rumen fluid was filtered through 4 layers of cheesecloth, then placed in pre-heated thermal flasks and immediately transported to the laboratory. Equal amounts of rumen contents from each animal were homogenized by agitation, and infused with nitrogen to maintain the anaerobic environment. The homogenized inoculum was stored in 5,000 mL Erlenmeyer flasks in a water bath at 39 °C. Following this, the rumen fluid was introduced into each fermenter until overflow occurred.

As recommended by Hoover et al. (1976), optimal fermentation conditions were maintained with a constant temperature of 39 °C, and continuous nitrogen infusion (40 mL/min). However, urea was added to the artificial saliva to mimic nitrogen recycling, and the pH was not controlled (Del Bianco Benedeti et al., 2015). Furthermore, the fermenter contents were agitated by a central propeller attached to a motor, responsible for homogenizing the fermenter contents. Fermenters were fed twice daily, at 0800 and 1600 hours. Fermenters receiving high- and low-concentrate diets were fed Fermenters were fed twice daily, equally divided in 2 meals/d, at 8 and 16 h. The artificial saliva consisted of sodium phosphate (Na_2_HPO_4_), sodium bicarbonate (NaCOH_3_), potassium chloride (KCL), magnesium sulfate heptahydrate (MgSO_4_7H_2_O), potassium bicarbonate (KHCO_3_), and urea (CH_4_N_2_O), dissolved in distilled water. Saliva and filtered fluid inflow rates were set to maintain solid and liquid flow rates of 5.5 and 11% per hour, respectively (Del Bianco Benedeti et al., 2015).

To determine concentrations of VFA and NH_3_–N, samples were collected from the 24-h pool on days 8, 9, and 10 of each incubation period. Samples taken from the 24-h pool were homogenized (liquid effluent and solid effluent), filtered through 4 layers of cheesecloth, and placed in 15 mL centrifuge tubes, pre-identified and containing 0.2 mL of H_2_SO_4_ solution (500 mL/L) for sample preservation. Subsequently, the samples were centrifuged in plastic tubes at 1,000 × *g* for 15 min at 4 °C. Following centrifugation, the supernatant was carefully extracted, transferred into 2 mL microtubes, and subjected to a second centrifugation at 20,000 × *g* for 30 min at 4 °C to prepare for subsequent VFA analysis.

The concentration and profile of VFA were determined by high-resolution gas chromatography using a gas chromatograph (Nexis GC-2030, Shimadzu) equipped with a Nukol capillary column (Supelco) measuring 30 m in length and 0.53 mm in diameter, coupled with a flame ionization detector (FID). The temperature was programmed to start at 100 °C and remain for 2 min, then increased to 130 °C at a rate of 10 °C/min. Subsequently, another temperature increases from 130 to 170 °C was carried out at 15 °C/min and maintained for 11 min. The injector and detector temperatures were set to 230 and 250 °C, respectively, and 0.5 µL samples were injected in “split” mode using nitrogen as the carrier gas.

For NH_3_–N analysis, samples were centrifuged at 1,000 × *g* for 15 min at 4 °C. After centrifugation, the supernatant was removed and stored in a 2 mL plastic microtube, then centrifuged again for 30 min at 20,000 × *g* at 4 °C. The concentration of NH_3_–N was determined by colorimetric analysis, as described by Chaney and Marbach (1962). Rumen nitrogen metabolism was determined by purine base quantification analysis, adapted from Ushida et al. (1985) and Zinn (1986).

Feed and digesta effluent samples were analyzed for DM, ash, neutral detergent fiber (NDF), ether extract (EE), and crude protein (CP) using INCT-CA methods (G-003/1, M-001/1, F-001/1, G-005/1, and N-001/1, respectively; Detmann et al. 2021). Organic matter (OM) was determined by subtracting ash content from DM. Samples of microbial pellet and digesta effluent background were assessed for DM, CP, and ash employing methods previously described for feed samples.

### Statistical Analysis

All results were tested for normality, Davis and Stephens (1989), and all followed a normal distribution (*P* > 0.05). All statistical procedures were performed using SAS 9.2 for Windows (Statistical Analysis System Institute, Inc., Cary, NC, USA) with α = 0.05. The data were analyzed using the SAS 9.4 mixed procedure (PROC MIXED; SAS, 2002) with a design of 2 simultaneous 5 × 5 Latin squares. Each Latin square featured 2 levels of concentrate (high and low) and 5 treatments. The model used is presented below:


$$\begin{array}{l} Y_{ijkl}=\alpha_{i}+\lambda_{j}+\alpha_{i}\lambda_{j}+P_{k}+A_{l}+e_{ijkl}, \end{array}$$

where *Y*_*ijkl*_ is the observed measurement of the *i*th level of concentrate in the diet and *j*th additive in the *k*th period and *l*th fermenter; *i* = 1, 2 (diet concentrate levels), *j* = 1 to 5 (additives), α_i_ = effect of the *i*th fixed qualitative factor (concentrate level, 2 levels); *λ*_*j*_ = effect of the *j*th additive; α_*i*_λ_*j*_ = interaction between concentrate levels and additives; *P*_*k*_ = effect of the *k*th level of the random factor period; *A*_*l*_ = effect of the level of the random factor fermenter; *e*_*ijkl*_ = unexplained residual error, assuming *e*_*ijk*_ ~ N(0, *s*²), with independent errors.

## Results

There was no interaction between concentrate levels and additives for any of the evaluated digestibility parameters (*P* > 0.05; Table 2). The DM and OM digestibility did differ statistically among concentrate levels (*P* < 0.01). Conversely, diets with high-concentrate levels showed lower CP (*P* = 0.01) and NDF (*P* < 0.01) digestibilities. There was no statistical difference among additives for DM (*P* = 0.10), OM (*P* = 0.22), and NDF (*P* = 0.25) digestibilities. However, treatments with Monensin, Blend 1, and Yeast Cells exhibited higher CP digestibility compared to Blend 2 (*P* < 0.01).

**Table 2.** Effects of inclusion of different additives in diets with 2 levels of concentrate on digestibility parameters in a dual-flow continuous culture system

Item^†^	Concentrate level^‡^		Additives^§^					SEM	*P*-value		
	Low	High	Control	Yeast cells	Blend 1	Blend 2	Monensin		Diet	Additive	Diet × Additive
Digestibility, g/kg											
Dry matter	508	704	593	595	636	572	632	27.1	<0.01	0.10	0.39
Organic matter	551	660	602	605	617	577	625	25.3	<0.01	0.22	0.91
Crude protein	821	689	711^bc^	787^ab^	770^ab^	695^c^	812^a^	38.2	0.01	0.04	0.37
NDF	622	323	456	472	508	433	492	28.0	<0.01	0.25	0.93

^a,b,c^Different letters on the same line indicate significant differences between means related to additives (*P* < 0.05).

^†^NDF = neutral detergent fiber; SEM = standard error of the mean.

^‡^High (80% concentrate), low (20% concentrate).

^§^Control—diet without additive inclusion; Yeast cells (*Saccharomyces cerevisiae*) hydrolyzed, inactivated, and dried by spray at 2,133 mg/kg DM (BioHydro Yessinergy LTDA, Campinas, SP, Brazil); Blend 1—yeast culture (*Saccharomyces cerevisiae*), beta-glucans, fructooligosaccharides, galactooligosaccharides, and mannanoligosaccharides at 1,600 mg/kg DM (Golf Yessinergy LTDA, Campinas, SP, Brazil); Blend 2—beta-glucan and mannanoligosaccharide fractions from *Saccharomyces cerevisiae* at 1,600 mg/kg DM (GlucanMos Yessinergy LTDA, Campinas, SP, Brazil); Monensin (Rumensin, 25 mg/kg DM).

Results for ruminal fermentation parameters are presented in Table 3. Total VFA concentration and butyrate concentration were higher for the high-concentrate diet (*P* < 0.01). Conversely, pH and concentrations of acetate and iso-butyrate were higher for the low-concentrate diet (*P* < 0.01). There was no statistical difference among additives for pH (*P* = 0.15), butyrate (*P* = 0.64), and iso-butyrate (*P* = 0.89) concentrations. However, treatments with Blend 1, Blend 2, and Yeast Cells had higher VFA concentrations compared to the control (*P* = 0.04). For acetate concentration, treatments with Blend 2, control, and Yeast Cells were superior to Blend 1 (*P* < 0.01).

**Table 3.** Effects of inclusion of different additives in diets with 2 levels of concentrate on fermentation parameters in a dual-flow continuous culture system

Item^†^	Concentrate level^‡^		Additives^§^					SEM	*P*-value		
	Low	High	Control	Yeast cells	Blend 1	Blend 2	Monensin		Diet	Additive	Diet × Addit
Total VFA, mmol	55.2	98.2	77.9^a^	74.0^b^	78.3^a^	77.7^a^	75.7^ab^	1.49	<0.01	0.04	0.66
VFA profile, mol/100 mol											
Acetate	72.6	46.7	60.3^a^	60.1^a^	58.5^b^	59.9^a^	59.4^ab^	0.46	<0.01	<0.01	0.77
Propionate	16.7	36.8	26.2^b^	26.2^b^	27.2^a^	26.9^ab^	27.1^a^	0.61	<0.01	0.02	<0.01
Butyrate	7.41	10.7	9.07	9.09	8.85	9.04	9.28	0.44	<0.01	0.64	0.74
Iso-butyrate	0.74	0.28	0.51	0.51	0.51	0.52	0.51	0.01	<0.01	0.89	0.28
Valerate	1.19	5.01	3.10^ab^	3.28^a^	3.16^ab^	2.95^c^	3.02^bc^	0.22	<0.01	0.01	<0.01
Iso-valerate	1.07	0.51	0.83^ab^	0.85^a^	0.76^c^	0.77^bc^	0.74^c^	0.55	<0.01	<0.01	<0.01
BCVFA	1.82	0.79	1.34^ab^	1.37^a^	1.27^bc^	1.29^bc^	1.25^c^	0.06	<0.01	0.01	<0.01
Acetate:propionate	4.37	1.28	2.90^a^	2.84^ab^	2.81^b^	2.81^b^	2.77^b^	0.65	<0.01	0.04	0.02
NH_3_–N, mg/100 mL	0.69	1.30	1.02^ab^	0.98^ab^	0.88^b^	1.09^a^	1.03^ab^	0.17	<0.01	0.17	0.04
pH	6.84	5.40	612	6.07	6.14	6.13	6.15	0.06	<0.01	0.15	0.56

^a,b,c^Different letters on the same line indicate significant differences between means related to additives (*P* < 0.05).

^†^VFA = Volatile fatty acids; BCVFA = Branched-chain volatile fatty acids; SEM = Standard error of the mean.

^‡^High (80% concentrate), low (20% concentrate).

^§^Control—diet without additive inclusion; Yeast cells (*Saccharomyces cerevisiae*) hydrolyzed, inactivated, and dried by spray at 2,133 mg/kg DM (BioHydro Yessinergy LTDA, Campinas, SP, Brazil); Blend 1—yeast culture (*Saccharomyces cerevisiae*), beta-glucans, fructooligosaccharides, galactooligosaccharides, and mannanoligosaccharides at 1,600 mg/kg DM (Golf Yessinergy LTDA, Campinas, SP, Brazil); Blend 2—beta-glucan and mannanoligosaccharide fractions from *Saccharomyces cerevisiae* at 1,600 mg/kg DM (GlucanMos Yessinergy LTDA, Campinas, SP, Brazil); Monensin (Rumensin, 25 mg/kg DM).

**Figure 1.**
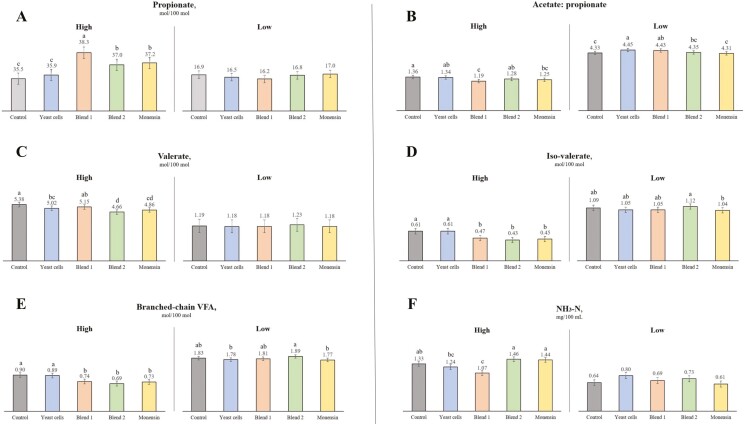
Effect of different additives on fermentation parameters in diets with high and low levels of concentrate in a dual-flow continuous culture system. ^abc^Different letters indicate significant differences among means (*P* < 0.05). *Control—diet without additive supplementation; Yeast cells (*Saccharomyces cerevisiae*) Hydrolyzed, inactivated, and spray-dried yeast cells (*S. cerevisiae*) at 2,133 mg/kg DM (BioHydro Yessinergy LTDA, Campinas, SP, Brazil); Blend 1—yeast culture (*S. cerevisiae*), beta-glucans, fructooligosaccharides, galactooligosaccharides, and mannanoligosaccharides at 1,600 mg/kg DM (Golf Yessinergy LTDA, Campinas, SP, Brazil); Blend 2—beta-glucans and mannanoligosaccharides fractions from *S. cerevisiae* at 1,600 mg/kg DM (GlucanMos Yessinergy LTDA, Campinas, SP, Brazil); Monensin (Rumensin, 25 mg/kg DM).

An interaction between concentrate levels and additives was observed for propionate, valerate, iso-valerate, branched-chain volatile fatty acids (BCVFA), acetate:propionate ratio, and NH_3_–N parameters (Figure 1). Specifically, the Blend 1 treatment exhibited significantly higher propionate concentrations in fermenters fed a high-concentrate diet (*P* < 0.01), whereas Control and Yeast Cells treatments showed the lowest values for this parameter (*P* < 0.01). In terms of valerate, Control and Blend 1 treatments had higher means compared to Blend 2 and Monensin (*P* < 0.01). Additionally, for iso-valerate in the high-concentrate diet, Control and Yeast Cells treatments had higher means than the other treatments (*P* < 0.01), while in low-concentrate diets, Blend 2 exhibited higher iso-valerate concentration compared to Monensin (*P* < 0.01). Regarding BCVFA in the high-concentrate diet, Control and Yeast Cells treatments had higher means than the other treatments (*P* < 0.01); similarly, in low-concentrate diets, Blend 2 showed a higher BCVFA concentration than Monensin and Yeast Cells (*P* < 0.01). Moreover, in the high-concentrate diet, Blend 1 exhibited a lower acetate: propionate ratio compared to Control, Yeast Cells, and Blend 2 treatments (*P* < 0.01); conversely, in the low-concentrate diet, the Yeast Cells treatment showed a higher acetate: propionate ratio compared to Control, Blend 2, and Monensin treatments (*P* < 0.01). Lastly, for NH_3_–N concentration in the high-concentrate diet, Blend 2 and Monensin treatments had higher values compared to Yeast Cells and Blend 1 treatments (*P* < 0.01).

Results for nitrogen metabolism parameters are presented in Table 4. There was no statistical difference between concentrate levels for NH_3_–N concentration (*P* = 0.41) and NH_3_–N and rumen undegradable protein (RUP) flows (*P* = 0.36 and *P* = 0.67, respectively). However, the high-concentrate diet showed higher values for all other parameters: Microbial efficiency (*P* = 0.03), Nitrogen (N) efficiency (*P* < 0.01), Cap N (*P* < 0.01), N flow (*P* < 0.01), non-ammonia N (NAN) flow (*P* < 0.01), and Bacterial N flow (*P* < 0.01). Treatments with Blend 2 and Control showed higher RUP-N flow compared to Yeast Cells and Blend 1 treatments (*P* < 0.01). There was no statistical difference between additives for the remaining nitrogen metabolism parameters (*P* > 0.05). There was no interaction between concentrate levels and additives for any of the evaluated nitrogen metabolism parameters (*P* > 0.05).

**Table 4.** Effects of inclusion of different additives in diets with 2 levels of concentrate on nitrogen metabolism parameters in a dual-flow continuous culture system

Item^†^	Concentrate level^‡^		Additives^§^					SEM	*P*-value		
	Low	High	Control	Yeast cells	Blend 1	Blend 2	Monensin		Diet	Additive	Diet × Addit
Microbial efficiency, g/kg	193	236	202	234	215	198	224	23.2	0.03	0.77	0.51
N efficiency, g/kg	477	701	548	639	581	536	641	58.0	<0.01	0.53	0.51
N capture, g/kg	385	593	454	531	483	444	534	47.8	<0.01	0.51	0.49
Flow, g											
N	1.07	1.62	1.35	1.35	1.29	1.39	1.32	0.07	<0.01	0.81	0.64
NH_3_–N	0.027	0.023	0.023	0.032	0.022	0.022	0.027	0.005	0.36	0.34	0.12
NAN	1.04	1.60	1.33	1.32	1.27	1.37	1.29	0.064	<0.01	0.81	0.70
Bacterial N	0.59	1.11	0.78	0.92	0.84	0.76	0.93	0.082	<0.01	0.43	0.41
RUP-N	0.45	0.49	0.55^ab^	0.39^c^	0.43^bc^	0.62^a^	0.36^c^	0.065	0.67	<0.01	0.50

^a,b,c^Different letters on the same line indicate significant differences between means related to additives (*P* < 0.05).

^†^N = nitrogen; NH_3_–N = ammoniacal nitrogen; NAN = non-ammonia nitrogen; RUP = undegradable protein.

^‡^High (80% concentrate), low (20% concentrate).

^§^Control—diet without additive inclusion; Yeast cells (*Saccharomyces cerevisiae*) hydrolyzed, inactivated, and dried by spray at 2,133 mg/kg DM (BioHydro Yessinergy LTDA, Campinas, SP, Brazil); Blend 1—yeast culture (*Saccharomyces cerevisiae*), beta-glucans, fructooligosaccharides, galactooligosaccharides, and mannanoligosaccharides at 1,600 mg/kg DM (Golf Yessinergy LTDA, Campinas, SP, Brazil); Blend 2—beta-glucan and mannanoligosaccharide fractions from *Saccharomyces cerevisiae* at 1,600 mg/kg DM (GlucanMos Yessinergy LTDA, Campinas, SP, Brazil); Monensin (Rumensin, 25 mg/kg DM).

## Discussion

The inclusion of concentrate directly impacts the rate and nature of fermentation, influencing ruminal dynamics and the composition of metabolic byproducts generated during this process (Lechartier and Peyraud, 2011). Consequently, notable differences in ruminal fermentation patterns are observed with diets containing varying concentrate levels (Huntington et al., 2006). Consistent with this reasoning, we expected to observe higher DM and OM digestibility with the high-concentrate diet, which indeed occurred. This could be attributed to the fermentative profile of the diet’s ingredients. Concentrate-rich feeds are characterized by their higher fermentative potential due to greater non-fibrous carbohydrate concentrations (Huntington et al., 2006). The microbial fermentation of these feeds leads to the production of VFA and a subsequent decrease in pH (Dias et al., 2018), which are events linked to increased digestibility of OM. Therefore, the higher total VFA concentration and OM digestibility values suggest greater fermentation efficiency with high-concentrate diets. The use of high levels of concentrate, especially with rapidly degradable starch sources, can lead to increased VFA production, improved digestibility, and consequently, greater metabolizable energy availability for the animal (Qiu et al., 2022).

Protein is a key nutrient in beef cattle nutrition, serving various roles in the animal’s body, such as tissue synthesis, enzyme and hormone production, and genetic composition (Samuelson et al., 2023). Factors like ruminal passage rate and pH can limit the availability and degradation of dietary proteins (Dias et al., 2018). In environments where pH remains below 6.0, the activity of protein-degrading organisms decreases, directly affecting dietary protein degradation (Iommelli et al., 2022). Thus, diets with high levels of rapidly fermentable carbohydrates can lower ruminal pH, significantly affecting protein degradation in the diet (Haryanto, 2014). This could explain the lower protein degradation observed in high-concentrate diets. Another consideration is the higher flow of RUP observed in this diet, potentially justifying lower protein degradation within the rumen environment.

The lower NDF digestibility observed in fermenters fed high-concentrate diets may be attributed to the less favorable conditions in the rumen for fibrolytic bacterial growth, particularly the reduced their ruminal pH (5.40) compared to those fed low-concentrate diets (6.84). This change leads to an increase in acid-adapted bacterial strains, such as *Streptococcus bovis*, *Selenomonas ruminantium*, and *Prevotella bryantii* (Pinloche et al., 2013), while fiber-fermenting bacteria like *Fibrobacter succinogenes*, *Ruminococcus albus*, and *Ruminococcus flavefaciens* experience reduced growth (Fernando et al., 2010). This alteration may negatively impact NDF digestibility in diets with high-concentrate levels.

The lack of difference in DM, OM, and NDF digestibility between monensin and other additives may suggest equivalence in their modes of action. The proven efficacy of sodium monensin as a growth promoter and performance optimizer in animals’ contrasts with the growing concern regarding potential antimicrobial resistance induction (Cuenca et al., 2022). Consequently, there is a growing interest in alternatives that preserve the benefits of monensin without associated risks. Research into natural additives like yeast-based additives that offer similar effectiveness has gained attention (Kogan and Kocher, 2007; Jia et al., 2018). Monensin and yeast-based prebiotics play distinct yet equally important roles in the ruminal environment. Monensin, being an antibiotic ionophore, modulates ruminal fermentation by selectively targeting bacteria, leading to changes in microbiota composition (Azzaz et al., 2015). This process results in favorable alterations in VFA production and nutrient utilization efficiency (Terry et al., 2019). Prebiotics, on the other hand, act as specific substrates for beneficial bacteria growth in the rumen (Miranda-Yuquilema et al., 2024), promoting a healthier microbiota, stimulating fibrolytic bacteria proliferation, and aiding in microbial flora balance maintenance (Mosoni et al., 2007). The comparable CP digestibility values observed among the Monensin, Blend 1, and Yeast Cells treatments suggest that these natural additives can enhance the ruminal environment, thereby improving nutrient degradation similarly to Monensin (Monnerat et al., 2013; Broadway et al., 2015). Conversely, the reduced CP digestibility in Blend 2 treatments may be associated with nitrogen balance. This additive also showed higher RUP-N flow, potentially affecting protein degradation within the ruminal environment (Refat et al., 2015).

Improving the ruminal environment by maintaining low oxygen levels, established by yeast-based additives, promotes viable bacteria growth and fiber-degrading bacteria (Bell et al., 2017). Thus, the absence of differences between additives in NDF digestibility could be explained by these additive mechanisms of action. In terms of ruminal fermentation parameters, a higher total concentration of VFA was expected in high-concentrate diets, which indeed occurred and correlates with the increased OM digestibility observed in this diet. The increased total ruminal population resulting from environmental improvements also contributes to higher VFA concentrations (Oeztuerk et al., 2005; Monnerat et al., 2013). In diets with higher concentrate content, the acetate: propionate ratio decreases due to the fermentative profile of these feeds (Robinson and Erasmus, 2009), which might explain the higher production of propionate, butyrate, and valerate, along with the lower acetate:propionate ratio observed in high-concentrate diets. The higher VFA concentration with yeast-based additives compared to the control was expected and suggests these additives’ potential to optimize dietary energy efficiency. By maximizing microbial growth, there is increased feed degradation, subsequently boosting VFA concentration (Dewhurst and Newbold, 2022). This indicates that yeast-based additives have the potential to replace sodium monensin commonly used in high-concentrate diets without negatively impacting ruminal fermentation parameters and VFA concentration.

Acetate production in the ruminal environment primarily stems from fibrous diet fermentation (Li et al., 2022). Yeast-based additives function by improving the ruminal environment, removing existing O_2_, directly benefiting fibrolytic bacteria (Vohra et al., 2016; Gharechahi and Salekdeh, 2018). This benefit was most noticeable in the roughage-rich diet, where the treatments with Blend 1 and Yeast Cells showed higher acetate: propionate ratios. Interestingly, Blend 1 exhibited a lower acetate: propionate ratio and a higher propionate concentration in high-concentrate diets. This suggests that the additive optimizes the fermentation of either fibrous or non-fibrous carbohydrates, depending on the diet type. This hypothesis is plausible given Blend 1’s unique composition, which includes GOS and FOS. The ability of GOS to increase propionate in the intestines of monogastric animals has been demonstrated (Smiricky-Tjardes et al., 2023). On the other hand, the combination of GOS and FOS may enhance fiber digestion and increase the production of total VFA, acetate, and butyrate (Kanakupt et al., 2011). Nevertheless, the increased propionate concentration can be beneficial to the animal, as propionate is the primary gluconeogenic precursor in ruminants (Hruby Weston et al., 2023).

Another favorable aspect of increased propionate is its potential to remove H^+^ from the ruminal environment, whereas acetate and butyrate are hydrogen producers, as pathways for acetate formation release 2 CO_2_ molecules and 8 hydrogens (Bell et al., 2017; Del Bianco Benedeti et al., 2018). In the rumen, hydrogen is primarily utilized for methane production, and the most efficient method to remove hydrogen is through methanogenesis (Greening et al., 2019). However, methane released during methanogenesis results in a considerable loss of provided gross energy, as the carbon lost in methane formation could potentially be utilized for lipid, carbohydrate, and amino acid synthesis (Valente et al., 2016).

The BCVFA are produced due to the oxidative deamination of amino acids such as valine, leucine, and isoleucine, derived from feed or ruminal microbial recycling (Reynolds and Kristensen, 2008). These compounds are important substrates for the growth of certain microorganisms responsible for degrading both fibrous and non-fibrous carbohydrates (An et al., 2023). This explains why the Blend 2 treatment showed higher iso-valerate and BCVFA values in low-concentrate diets while exhibiting lower values of these parameters in high-concentrate diets compared to the control group.

Valerate primarily derives from the fermentation of fibrous and protein components within the diet (Gharechahi and Salekdeh, 2018). In high-concentrate diets, treatments with Yeast Cells, Blend 2, and Monensin exhibited reduced NDF and CP digestibility along with lower valerate concentration. The decreased NDF content in high-concentrate diets, as opposed to diets containing 80% Tifton 85 hay, explains the observed decline in valerate concentration.

Ruminal NH_3_–N concentrations are influenced by the degradation of dietary protein fractions, with decreases in ruminal protein oxidation leading to lower NH_3_–N levels (Titze et al., 2023). This could explain the observed lower NH_3_–N concentration in the Blend 1 treatment specifically in high-concentrate diets. Additionally, during fermentation, dietary protein is largely converted into carbon chains and NH_3_–N (Titze et al., 2023), which serves as a substrate for microbial growth. Therefore, the lack of significant additive effects on microbial efficiency suggests that yeast-based additives did not negatively interfere with the utilization of dietary nitrogen by ruminal microorganisms.

Our hypothesis was that yeast-based additives could serve as a replacement for sodium monensin without affecting nitrogen metabolism. Indeed, the use of natural additives did not negatively impact the evaluated parameters related to this topic. However, we observed a lower flow of rumen RUP nitrogen for the yeast cell and monensin treatments, which correlates with CP digestibility in these treatments. Maintaining a balance between ruminal degradable protein and RUP in the diet is crucial for ensuring optimal animal performance (Molosse et al., 2023). Ruminant protein requirements are fulfilled by amino acids absorbed in the small intestine, derived from RUP and microbial protein (Mariz et al., 2018). Meeting the metabolizable protein needs of medium to high-performance animals is essential for maximizing productivity (Molosse et al., 2023). Even with adequate dietary protein concentration, maximizing microbial protein production offers an alternative approach to achieving this goal (Qiao et al., 2010). These findings suggest that the Blend 2 treatment could be beneficial for high-performance animals with elevated protein requirements.

## Conclusion

Our findings demonstrate that incorporating yeast-based additives into the tested diets yielded promising results compared to the traditional use of sodium monensin. In diets with high levels of concentrate, yeast-based additives (Blend 1) enhanced CP digestibility, increased valerate concentration, and elevated propionate levels and the acetate: propionate ratio. These results suggest that this additive could improve energy efficiency, reducing substrate availability for CH_4_ formation, which benefits both the performance and sustainability of feedlot-fed cattle.

In diets with high forage inclusion, Blend 2 increased acetate levels, nitrogen flux RUP, as well as iso-valerate and BCVFA levels. These effects indicate that this additive could enhance nutrient utilization in pasture-raised animals, offering economic and productive benefits in beef production systems. Therefore, in vivo studies are needed to confirm the results observed in the present study.

## Abbreviations:

BCVFA: branched-chain volatile fatty acids
CP: crude protein
DM: dry matter
EE: ether extract
N: nitrogen
NDF: neutral detergent fiber
NH3–N: ammonia nitrogen
OM: organic matter
RUP: rumen undegradable protein
VFA: volatile fatty acids
